# Toward the Abandonment of Axillary Surgery: Is This the End of an Era?

**DOI:** 10.7759/cureus.107526

**Published:** 2026-04-22

**Authors:** Maria Papadoliopoulou, Alexios Kozonis, Ioannis Margaris

**Affiliations:** 1 4th Department of Surgery, Attikon University Hospital, University of Athens, Athens, GRC; 2 Department of Obstetrics and Gynaecology, Attikon University Hospital, University of Athens, Athens, GRC

**Keywords:** axillary surgery, breast cancer, imaging/spectroscopic techniques, omission of slnb, sentinel lymph node biopsy (slnb)

## Abstract

Axillary surgery in breast cancer has evolved substantially from radical surgical approaches toward minimally invasive and, in selected cases, non-surgical strategies.

This editorial summarizes emerging techniques for axillary staging, with particular emphasis on advanced imaging and optical spectroscopic methods, including contrast-enhanced ultrasonography, Raman spectroscopy, optical coherence tomography, Fourier-transform infrared spectroscopy, elastic scattering spectroscopy, and refractometry. These modalities demonstrate promising diagnostic accuracy and may allow for preoperative or intraoperative axillary staging, obviating the need for surgical excision.

At the same time, ongoing randomized trials are investigating the omission of sentinel lymph node biopsy in carefully selected patients with early-stage breast cancer and clinically negative axillary lymph nodes. Early results suggest oncological safety in low-risk populations, although longer follow-up data are required.

These developments suggest a future of personalized axillary management, potentially marking the beginning of the end for routine axillary surgery in breast cancer care.

## Editorial

More than 200 years ago, William S. Halsted first described axillary lymph node dissection (ALND), a procedure that provided local control and contributed to improved overall survival rates in patients with breast cancer. The latter remained the “gold standard” technique until 1971, when Fisher et al., in their seminal NSABP B-04 trial, suggested that ALND was not therapeutic but mostly a staging tool and could be substituted by radiotherapy for obtaining local control [1]. In 1997, Veronesi et al. introduced the axillary sentinel lymph node biopsy (SLNB) technique, which had been previously described by Morton et al. in patients with melanoma [2]. Since then, SLNB has remained the standard of care for patients with preoperative clinically negative axilla [2].

However, evolution is continued in axillary surgery, with a ‘less is more’ approach. Many authors underline a shift from more extensive to more targeted and minimalistic manners in the evaluation of the status of axillary lymph nodes in patients with breast cancer [3]. Thus, trending research of the future is focused on two different pathways: (1) preoperative staging of the axilla with imaging techniques or intraoperative staging with spectroscopic techniques without the need for surgical resection, and (2) omission of SLNB in selected patients with early breast cancer.

In this editorial, a brief review of changing trends in axillary surgery is presented.

Imaging techniques

Ultrasonography is commonly used for preoperative axillary evaluation but demonstrates a moderate sensitivity of approximately 50-60% and a high specificity of 97-100% [4]. Breast and axillary magnetic resonance imaging presents a high specificity as well (90-100%) and improved sensitivity (73-94.6%) compared to ultrasonography [5]. Computed tomography (CT) and positron- emission tomography/CT suffer from limited sensitivity and a substantial false-negative rate [6].

Small studies have evaluated the use of contrast-enhanced ultrasonography in the evaluation of axillary lymph node status. In a recent study including 42 patients, the microvasculature of axillary lymph nodes was evaluated by using a contrast agent and a high-resolution probe. The contrast enhancement pattern differed between benign and malignant nodes, with a statistical difference (p<0.05) [7].

In a preliminary study published in 2024, a microscope integrating autofluorescence imaging with Raman spectroscopy was used to scan samples of axillary lymph nodes. A total of 85 samples, both healthy and cancerous, were analyzed, and the results were very promising. The area under the curve was 0.93 (0.83-0.98) with an accuracy of up to 94% [8].

Alternative tools for axillary lymph node assessment are the spectroscopic/imaging techniques such as elastic scattering spectroscopy (ESS), optical coherence tomography (OCT), Raman spectroscopy, Fourier-transform infrared spectroscopy (FTIR), and refractometry. They encompass an emerging field that is closely related to cancer research. Optical imaging uses light properties to obtain detailed images of organs, tissues, cells, and even molecules, showing promising results in early disease detection, diagnosis, and treatment response monitoring [9,10].

ESS is a low-cost and consistent method for intraoperative diagnosis of breast cancer lymph node metastasis with adequate sensitivity and specificity, 76-84% and 91-96%, respectively. OCT provides adequate tissue scanning, along with a short data acquisition time, offering real-time diagnosis. Raman spectroscopy proves to be a spectroscopic method of high accuracy for the identification of axillary lymph node involvement, with reported sensitivity and specificity values of 71-81% and 97%, respectively. In addition, FTIR is a promising method with a high sensitivity (94.7%), a high specificity (90%), and a rapid acquisition time of 2-3 minutes, but these results are yet to be confirmed from larger studies. Lastly, a recent study on normal and malignant lymph nodes using a prism-coupled refractometer found that the median refractive indices were significantly higher in cancerous lymph nodes than in normal lymph nodes at 450 nm, 964 nm, and 1551 nm wavelengths (p<0.05) [9,10].

Overall, all of the aforementioned experimental spectroscopic/imaging studies have shown promising results for early disease detection, diagnosis, and treatment response monitoring. 

Omission of axillary surgery

The first attempt to evaluate the omission of SLNB was a side observation in the Cancer and Leukemia Group B (CALGB) 9343 trial, in which the use of radiotherapy was evaluated in patients with early breast cancer (T1, estrogen-positive, aged over 70 years old). Randomized patients in both arms had not received any axillary surgery in a significant percentage (60%), and no significant difference in overall survival or breast-cancer-specific survival was observed. Those results were followed by the question whether that was valid for all age groups with early breast cancer [11].

The omission of axillary surgery is investigated in terms of oncological safety in several pilot trials. No Axillary sUrgical Treatment In cLinically lymph node negative UltraSonography (NAUTILUS) trial is an ongoing prospective multicenter randomized trial, in which T1-2 patients with breast cancer treated with breast-conserving therapy and preoperative ultrasonography negative for suspicious lymph nodes are equally randomized to perform or omit SLNB. The results of this study are pending [12]. Similar ongoing trials evaluating T1-2 breast cancer with preoperative negative axilla and planned breast-conserving surgery are BOAG 13-08 and SOAPET.

Results of the Sentinel node vs Observation after axillary UltrasouND (SOUND) trial have firstly been published. The study included patients who had T1 breast cancer, breast-conserving surgery, and planned whole-breast radiation. Five-year distant disease-free survival was equally great between the SLNB group and the no axillary surgery group (97.7% vs 98%, non-inferiority, p=0.02). Given the relatively short follow-up (5.7 years) and the fact that the majority of patients had ER-positive, HER2-negative breast cancers, the study underwent criticism regarding the possibility of late recurrences [13]. More recently another trial on omission of axillary surgery published its primary results. This is Intergroup-Sentinel-Mamma (INSEMA) trial that studied T1 but also a percentage of patients with larger tumors (T2) and announced that the omission of surgical axillary staging was non-inferior to sentinel-lymph-node biopsy after a median follow-up of six years [6,14].

Conclusion

Axillary surgery in patients with breast cancer has undergone a profound transformation over the past two centuries, evolving from radical dissection to increasingly conservative and individualized approaches. Current evidence supports the paradigm shift of “less is more,” driven by the dual goals of maintaining oncologic safety while minimizing morbidity. Advances in spectroscopic/imaging techniques offer promising alternatives for axillary staging, with several modalities demonstrating high diagnostic accuracy and the potential for real-time, non-invasive or intraoperative assessment without surgical excision. Although most of these technologies remain experimental, their continued development may significantly reduce the need for surgical staging in the future.

On the other hand, in carefully selected patients with early-stage breast cancer and clinically negative axillary lymph nodes even the need for staging is questioned. Omission of axillary surgery is being actively investigated in large randomized trials. Early results, particularly from the SOUND trial, suggest that avoiding SLNB may be oncologically safe in low-risk populations, although longer follow-up data are required to confirm the outcomes.

Overall, the future of axillary management appears to lie in tailored strategies that integrate diagnostic technologies with careful patient selection. Ongoing trials and technological innovation are expected to further change the role of axillary surgery, moving toward less invasive, yet equally effective, approaches in breast cancer care.

## References

[1] Fisher B, Jeong JH, Anderson S, Bryant J, Fisher ER, Wolmark N (2002). Twenty-five-year follow-up of a randomized trial comparing radical mastectomy, total mastectomy, and total mastectomy followed by irradiation. N Engl J Med.

[2] Veronesi U, Paganelli G, Galimberti V (1997). Sentinel-node biopsy to avoid axillary dissection in breast cancer with clinically negative lymph-nodes. Lancet.

[3] Dumitru D, Khan A, Catanuto G, Rocco N, Nava MB, Benson JR (2018). Axillary surgery in breast cancer: the beginning of the end. Minerva Chir.

[4] Le Boulc'h M, Gilhodes J, Steinmeyer Z, Molière S, Mathelin C (2021). Pretherapeutic imaging for axillary staging in breast cancer: a systematic review and meta-analysis of ultrasound, MRI and FDG PET. J Clin Med.

[5] Kuijs VJ, Moossdorff M, Schipper RJ (2015). The role of MRI in axillary lymph node imaging in breast cancer patients: a systematic review. Insights Imaging.

[6] Huang T, Wang W, Sun X (2025). Sentinel lymph node biopsy omission in early-stage breast cancer: current evidence and clinical practice. Front Oncol.

[7] Jain R, Khanna R, Verma A, Mishra SP, Meena RN, Khanna S, Khanna S (2024). Evaluation of axillary lymph nodes in breast cancer patients with clinically negative axilla using contrast enhanced ultrasonography. World J Surg Oncol.

[8] Barkur S, Boitor RA, Mihai R (2024). Intraoperative spectroscopic evaluation of sentinel lymph nodes in breast cancer surgery. Breast Cancer Res Treat.

[9] Papadoliopoulou M, Matiatou M, Koutsoumpos S (2023). Optical imaging in human lymph node specimens for detecting breast cancer metastases: a review. Cancers (Basel).

[10] Papadoliopoulou M, Koutsoumpos S, Margaris I (2025). Real index of refraction of normal and cancerous axillary lymph nodes in breast cancer patients: results from an experimental study. J Pers Med.

[11] Hughes KS, Schnaper LA, Bellon JR (2013). Lumpectomy plus tamoxifen with or without irradiation in women age 70 years or older with early breast cancer: long-term follow-up of CALGB 9343. J Clin Oncol.

[12] Chang JM, Shin HJ, Choi JS (2021). Imaging protocol and criteria for evaluation of axillary lymph nodes in the NAUTILUS trial. J Breast Cancer.

[13] Gentilini OD, Botteri E, Sangalli C (2023). Sentinel lymph node biopsy vs no axillary surgery in patients with small breast cancer and negative results on ultrasonography of axillary lymph nodes: the SOUND randomized clinical trial. JAMA Oncol.

[14] Reimer T, Stachs A, Veselinovic K (2025). Axillary surgery in breast cancer - primary results of the INSEMA trial. N Engl J Med.

